# ViWrap: A modular pipeline to identify, bin, classify, and predict viral–host relationships for viruses from metagenomes

**DOI:** 10.1002/imt2.118

**Published:** 2023-06-07

**Authors:** Zhichao Zhou, Cody Martin, James C. Kosmopoulos, Karthik Anantharaman

**Affiliations:** ^1^ Department of Bacteriology University of Wisconsin–Madison Madison Wisconsin USA; ^2^ Microbiology Doctoral Training Program University of Wisconsin–Madison Madison Wisconsin USA

**Keywords:** metagenome, microbiome, phage, virome, viruses

## Abstract

Viruses are increasingly being recognized as important components of human and environmental microbiomes. However, viruses in microbiomes remain difficult to study because of the difficulty in culturing them and the lack of sufficient model systems. As a result, computational methods for identifying and analyzing uncultivated viral genomes from metagenomes have attracted significant attention. Such bioinformatics approaches facilitate the screening of viruses from enormous sequencing data sets originating from various environments. Although many tools and databases have been developed for advancing the study of viruses from metagenomes, there is a lack of integrated tools enabling a comprehensive workflow and analysis platform encompassing all the diverse segments of virus studies. Here, we developed ViWrap, a modular pipeline written in Python. ViWrap combines the power of multiple tools into a single platform to enable various steps of virus analyses, including identification, annotation, genome binning, species‐ and genus‐level clustering, assignment of taxonomy, prediction of hosts, characterization of genome quality, comprehensive summaries, and intuitive visualization of results. Overall, ViWrap enables a standardized and reproducible pipeline for both extensive and stringent characterization of viruses from metagenomes, viromes, and microbial genomes. Our approach has flexibility in using various options for diverse applications and scenarios, and its modular structure can be easily amended with additional functions as necessary. ViWrap is designed to be easily and widely used to study viruses in human and environmental systems. ViWrap is publicly available via GitHub (https://github.com/AnantharamanLab/ViWrap). A detailed description of the software, its usage, and interpretation of results can be found on the website.

## Highlights

- ViWrap integrates state‐of‐the‐art tools and databases for the comprehensive characterization and study of viruses from metagenomes and genomes.
- ViWrap offers a highly flexible, modular, customizable, and easy‐to‐use pipeline with options for various applications and scenarios.
- ViWrap enables a standardized and reproducible pipeline for viral metagenomics, genomics, ecology, and evolution.

## Graphical abstract

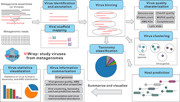

The rapidly growing repertoire of metagenomic/viromic assemblies from various ecosystems, including natural environments, industrial man‐made environments, and human‐microbiome‐related environments, has provided valuable sources for mining viral diversity. Since 2016, scientists have greatly enriched the collection of viruses in public databases and have advanced our understanding of viruses in nature by using uncultivated viral genomes obtained from metagenomes [1]. This has led to important discoveries that viruses have significant roles in reshaping microbial host metabolism and driving global biogeochemical cycles [1, 2]. Viruses encode auxiliary metabolic genes (AMGs) that augment host functions, typically for the benefit of the virus [3, 4]. These AMGs can maintain, drive, or short‐circuit important metabolic steps and provide viruses with fitness advantages [4, 5]. Given the discovery of many uncultivated viral genomes and their AMGs, scientists have unraveled their involvement in significant ecological functions, including photosynthesis [6, 7, 8], methane oxidation [9], sulfur oxidation [10, 11, 12], ammonia oxidation [13], ammonification [14], carbohydrate degradation [15, 16, 17], and other functions. In spite of these advances, our understanding of viruses continues to lag behind bacteria and archaea primarily due to the lack of available tools to study and advance viral ecology. This calls for a greater focus on the development of computational techniques facilitating virus analysis from microbiomes with a focus on metagenomic and metatranscriptomic data.

Recovering uncultivated viral genomic sequences typically involves one of two approaches, that is, their recovery either from bulk metagenomes or viromes. Bulk metagenomes include all genetic materials of the microbial community, and viral fractions only account for a small portion of bulk metagenomes. Viromes, on the other hand, represent enriched and concentrated viral fractions and exclude other members of the microbial community. Many tools have been developed for virus identification from both bulk metagenomes and viromes. For example, Microseek uses protein similarity to detect viruses [18]. It achieves this by scoring the lowest common ancestor of translated reads and contigs against a reference database of viral proteins [18]. VIP (Virus Identification Pipeline) aligns both nucleotide and amino acid sequences to the Reference Sequence (RefSeq) reference database to get viral reads and then assemble viral contigs; additionally, it also provides downstream analyses of taxonomy identification, coverage, and phylogenetic analysis [19]. Among all the currently available tools, VIBRANT, VirSorter2, and DeepVirFinder are three popular software for the identification of viruses from bulk metagenomes and viromes. VIBRANT uses a hybrid machine learning and protein similarity approach for automated recovery and annotation of viruses [20]. VirSorter2 uses a collection of customized automatic classifiers to achieve high virus recovery performance [21]. DeepVirFinder trains viral *k*‐mer‐based machine‐learning classifiers to identify viruses [22].

Post virus identification, software and approaches have been developed for virus genome binning, identification of viral taxonomy, determination of genome completion estimates, and prediction of hosts of viruses. vRhyme bins viral genomes by using both the coverage effect size and nucleotide features of viral scaffolds [23]. vConTACT2 uses whole genome gene‐sharing networks for distance‐based hierarchical clustering and prediction of viral taxonomy [24]. dRep enables virus clustering by dereplicating genomes based on sequence identity [25]. CheckV enables checking the quality and completeness of viral genomes [26], and iPHoP integrates all currently available virus–host relationship prediction methods and builds a machine‐learning framework to obtain comprehensive host predictions for viruses [27]. Beyond these tools, multiple previously curated virus databases contain protein sequences that can be used to guide virus taxonomy classification. For example, National Center for Biotechnology Information (NCBI) RefSeq stores reference viral genomes [28], VOGDB provides preclustered viral markers of viral orthologous groups hidden Markov models (VOG HMMs) (http://vogdb.org), and the IMG/VR v4 database (currently the largest virus‐specific genomic database) has high‐quality viral operational taxonomic units (vOTUs) with taxonomy preassigned by stringent methods [29, 30]. Nevertheless, these tools and databases are being used increasingly, serving as individual links within a large and complex chain of different software and approaches that are needed for comprehensive analyses of viral diversity and ecology. Given the relative infancy of the field of viromics, the knowledge of which tools to use, how to integrate methods, and to interpret results is often difficult for users with limited familiarity with viruses and bioinformatic skills. An integrated pipeline that covers the entire workflow of analyses of viruses and provides easy‐to‐read/parse results would significantly advance the field of virology and democratize the study of viruses from metagenomes and microbiomes. Additionally, this integrated pipeline can be seamlessly linked to advanced downstream viral evolutionary analysis pipelines, such as MetaPop [31], providing an enhanced capacity to investigate the evolutionary dynamics of viral genomes.

To address this problem, we have developed ViWrap, an integrated and user‐friendly modular pipeline to study viral diversity and ecology. ViWrap can identify, bin, classify, and predict virus–host relationships for viruses from metagenomes. It integrates the following advanced approaches: (1) a comprehensive screening for viruses while still keeping stringent rules; (2) a standardized and reproducible pipeline that integrates advanced tools/databases and is easy to amend for additional functionalities in the future; (3) flexible options for identifying methods, using metagenomic reads (with or without reads; short or long reads), and custom microbial genomes for various application scenarios; and (4) a one‐stop workflow to generate easy‐to‐read/parse results with visualization and statistical summary of viruses in samples. ViWrap will significantly simplify the current computational routine to study viruses from metagenomes, speed up research in screening more viral diversity from newly generated or previously deposited metagenomes/viromes, and promote the understanding of viral community structure and function in environmental and human microbiomes.

## METHODS

**Figure 1:**
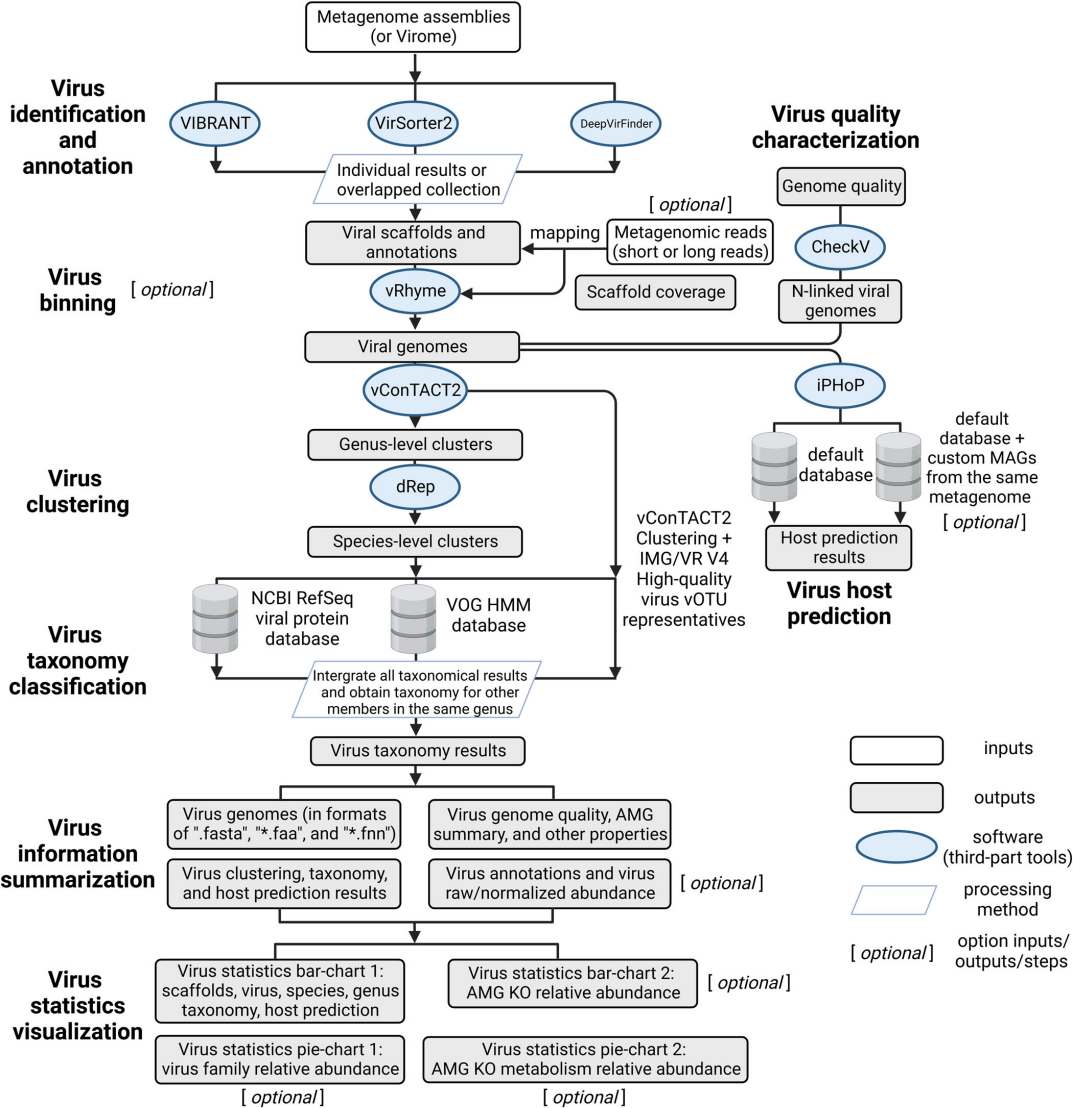
Flowchart describing the different steps and functionalities in ViWrap. Empty squares indicate inputs, filled squares indicate outputs, ovals indicate software, and parallelograms indicate the processing method that was used to get downstream results.

ViWrap is a pipeline/wrapper to integrate several popular virus analysis software/tools to identify, bin, classify, and predict virus–host relationships from metagenomes. It takes advantage of diverse software/tools to integrate them into a modular pipeline to obtain comprehensive information on virus genomics, ecology, and diversity in a user‐friendly way. ViWrap has eight different functionalities for virus analysis, including “Virus identification and annotation” (by VIBRANT, VirSorter2, and DeepVirFinder), “Virus binning” (by vRhyme), “Virus clustering” (by vConTACT2 to the genus level and dRep to the species level), “Virus taxonomy classification” (by NCBI RefSeq viral protein database, VOG HMM database, and IMG/VR v4 database [29, 30]), “Virus information summarization,” “Result visualization,” “Virus quality characterization,” and “Virus host prediction” (by iPHoP). The intended inputs are metagenome assemblies or viromes alongside metagenomic reads. Here, we define metagenome assemblies as assemblies reconstructed from bulk metagenomes containing mixed communities of prokaryotes, eukaryotes, and viruses, and viromes as assembled sequences from filtered/concentrated virion DNA in which viruses account for a dominant portion. Reads from metagenomes and viromes are referred to as metagenomic reads throughout the rest of the manuscript. The outputs are user‐friendly tables and figures, including virus genomes and associated statistics, clustering, taxonomy, host prediction results, annotation and abundance results, and a corresponding visualization of statistical summary (details described in Figure 1).

ViWrap can be used in conjunction with or without metagenomic reads, although using reads provides advantages and enables certain analyses. Specifically, to further facilitate using metagenomes/viromes/genomes for virus mining with the corresponding metagenomic reads unavailable, we introduced a specific “run_wo_reads” Python task. ViWrap is able to solely intake metagenomes/viromes or genomes without the input of metagenomic reads. When applying this task, ViWrap will avoid the steps of metagenomic mapping and virus binning, thus only reporting the results for viruses at the resolution of single scaffolds without the context of genome bins. Additionally, we implemented “set_up_env” and “download” tasks for downloading and setting up the conda environments and databases in a single step. To save on storage space required by the final result folders, we introduced a “clean” task to clean redundant information in each result directory.

ViWrap is written in Python and needs conda environments to achieve proper performance. The software is deposited in GitHub (https://github.com/AnantharamanLab/ViWrap). Details of the program's description, installation, running methods, and explanations of inputs and outputs can be found on the GitHub page. An example ViWrap run was conducted on a metagenome data set using the metagenomic assembly and reads of a microbial community inhabiting the deep‐sea hydrothermal vent environment of Guaymas Basin in the Pacific Ocean [32]. To enable ease of use for users looking to use this as a test data set with a shorter running time, we used a subset of the assembly (18,000 scaffolds, ~10% of total) and two subsets of the original reads with 10% and 15% of the total reads (randomly picked), respectively, as the inputs. Additionally, 98 previously reconstructed metagenome‐assembled genomes (MAGs) from the same data set were used for virus–host prediction by iPHoP based on custom host genomes.

## RESULTS

### Workflow of ViWrap

**Figure 2:**
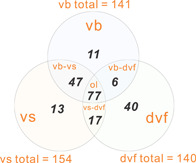
Venn diagram representing the overlapped viral scaffolds (intersection) identified by three methods. The results of individual methods were adopted from the demonstration of the example metagenome data set of the Guaymas Basin hydrothermal vent sample. dvf, DeepVirFinder; ol, overlapped viral scaffolds by “vb,” “vs,” and “dvf”; vb, VIBRANT; vs, VirSorter2; vb–dvf, VIBRANT and DeepVirFinder; vb–vs, VIBRANT and VirSorter2; vs–dvf, VirSorter2 and DeepVirFinder.

The detailed workflow of ViWrap is described in Figure 1. First, ViWrap can take metagenomic assemblies or viromes as the input source to identify viruses. Three methods were integrated to identify viral scaffolds using different algorithms, namely VIBRANT, VirSorter2, and DeepVirFinder. Results of virus identification are generated using methods of a user's choice, namely, either individual results from a single identification method (i.e., VIBRANT, VirSorter2, or DeepVirFinder) or combined results by taking the intersection of results of different identification methods (i.e., VIBRANT–VirSorter2 or VIBRANT–VirSorter2–DeepVirFinder). These three methods have different accuracy and performance in identifying viruses. As benchmarked by a study using these three tools and seven other tools in identifying viruses from metagenomes, all these three tools have excellent performance in dealing with artificial RefSeq contigs; the precision, recall, and F1 scores are also above 0.85 [33]. Using gene‐based methods, VIBRANT and VirSorter2 have high accuracy for diverse viruses, significantly facilitating the detection of novel viruses outside the groups most represented in the current reference databases [20, 21]. VIBRANT uses a machine‐learning neural network algorithm and quantitative v‐score metric to maximize the identification of highly diverse viruses [20]. VirSorter2 uses a collection of customized automatic classifiers to improve both the detection range and accuracy of viruses; it has high specificity in minimizing classification errors introduced by eukaryotic genomes and plasmids [21]. Both these two tools are state‐of‐the‐art tools for virus identification. Contrastingly, DeepVirFinder is currently one of the best tools using *k*‐mer‐based methods to predict viral sequences [22, 33]. However, all *k*‐mer‐based methods depend on virus reference databases which are limited by being biased toward viruses in RefSeq and other isolated viruses, and do not account for divergent viruses that can be recovered from metagenomic studies. Hence, a common issue for all *k*‐mer‐based methods is reference bias, which negatively impacts algorithms to identify novel and diverse viruses from metagenomes [33]. Therefore, we used the “VIBRANT–VirSorter2” method as the default approach to generate a comprehensive yet stringent viral scaffold collection that meets the requirements of two popular virus identification methods (Figure 2). This “VIBRANT–VirSorter2” combination method is a recommended setting for users. The benchmarking results of individual viral scaffold‐identifying methods can be found in previous publications [20–22, 33], but here, we do not provide any benchmarking results for either single or combined viral scaffold‐identifying methods. The AMG identification and viral protein annotations were parsed based on the result of VIBRANT. Specifically, the AMG identifications presented here are based on the AMG KEGG orthologs (KO) collection provided by VIBRANT and should be considered preliminary. Manual validation is necessary to ensure a more reliable and accurate result.

In the second step, metagenomic reads are used to map onto the given metagenomic assemblies or viromes to get the scaffold coverage. Both short reads and long reads can be used as inputs to generate scaffold coverage results. The scaffold coverage file is used to bin viral genomes by vRhyme. To achieve stringent criteria to assign viral scaffolds into a given viral bin (viral genome), we have adopted the following requirements: (1) In vRhyme settings, the maximum protein redundancy of a viral genome was set to 5; (2) a viral scaffold that was discovered to be a “Complete” virus by CheckV is not assigned to a viral genome; (3) a bin with one or more lytic members and one integrated provirus will not be considered and will be split; (4) a bin with two or more lysogenic members (including both lysogenic scaffolds and integrated proviruses) will not be considered and will be split. Finally, CheckV is used to estimate the genome qualities of all viruses identified. Due to the fact that CheckV requires a single‐scaffold virus as input, multiple‐scaffold viral genomes were linked by multiple Ns to meet the requirement. However, because the order of linking affects open reading frame (ORF) prediction, and some ORFs would not be called due to Prodigal's stringency in predicting ORFs as it gets closer to the Ns junctions, these N‐linked multiple fasta files are only used for estimating genome qualities by CheckV. Additionally, CheckV was not able to detect contamination from different viruses, so the completeness result assigned for the multiple‐scaffold virus cannot rule out the potential contaminations from other viral species.

In the third step, genus‐level clusters (viral genera) are classified by vConTACT2 (genomes within the same “VC subcluster” are regarded as from the same genus), and species‐level clusters (viral species) are classified by dRep (genomes with average nucleotide identity > 0.95 and alignment fraction > 0.85 are regarded as from the same species; please be aware that the viral species described here may not adhere to the “new reference species” proposed by the MIUiVG [34]). Currently, vConTACT2 has not been tested or validated for eukaryotic viruses [24]; it is suggested only to apply this on prokaryotic metagenomes when using ViWrap.

In the fourth step, three methods are used to assign taxonomy to each virus. Two of these include protein searches using the NCBI RefSeq viral protein database and HMM marker proteins in the VOG database based on instructions described previously [30]. For the third method, we use the vOTU representatives from IMG/VR v4 high‐quality vOTUs as anchors in individual genus‐level clusters assigned by vConTACT2 in the previous step to assign the taxonomy information. Finally, we integrate all these three taxonomic results. When one virus has multiple taxonomic results from these three methods, the final result is provided by following the priority order of the NCBI RefSeq viral protein search method, the VOG HMM marker search method, and the vConTACT2 clustering method. To obtain the taxonomy of viruses unassigned by any of these three methods, we first enter into each genus to determine if any virus genomes have already been classified using the NCBI RefSeq viral protein search method (only the hits from this classification method will be counted) and then expand the taxonomy to all members within the genus.

In the fifth and final step, we use iPHoP to predict hosts for viruses. Both the default iPHoP database and custom MAGs from the same metagenome can be used for host prediction. Using custom MAGs from the same metagenome can facilitate establishing direct connections between viruses and MAGs from the same community.

Finally, virus information, including comprehensive virus and AMG summary, is presented, and statistics are visualized accordingly.

### Layout of results

The resulting folders and files are arranged in the final output directory in the following order:

*00_VIBRANT_VirSorter_input_metageome_stem_name*: Result of the virus identification step. This folder contains the result folders of both VIBRANT and VirSorter2 runs; additionally, a folder containing the combined results of both runs is also provided. The annotation files, “fasta” (nucleotide sequence) file, “ffn” (gene sequence) file, and “faa” (protein sequence) file, are provided for viruses in the combined results.

*01_Mapping_result_outdir*: Result of the read mapping step. Both the raw scaffold coverage result generated by CoverM (https://github.com/wwood/CoverM) and the converted coverage result used as vRhyme input are provided in the folder.

*02_vRhyme_outdir*: Result of genome binning using vRhyme. The directory contains the folders “vRhyme_best_bins_fasta,” “vRhyme_best_bins_fasta_modified” (the best bins that were modified by stringent criteria described above), and “vRhyme_unbinned_viral_gn_fasta” (the unbinned viral scaffolds regarded as single‐scaffold viruses). Additionally, it contains two tables representing the lytic/lysogenic state of viruses and genome completeness information for viruses in the “vRhyme_best_bins_fasta” folder.

*03_vConTACT2_outdir*: Result of classification using vConTACT2. The directory contains combined protein and virus clustering results for both viruses identified from the above steps and the vOTU representatives from IMG/VR v4 high‐quality vOTUs.

*04_Nlinked_viral_gn_dir*: N‐linked viral genomes used as CheckV inputs. The directory contains viral genomes with all scaffolds linked by multiple Ns. Here, only for meeting the requirement of input file format for CheckV, single‐scaffold viruses (N‐linked or originally single‐scaffold) are used.

*05_CheckV_outdir*: Result of CheckV analyses. The directory contains individual CheckV result folders for each virus and the summarized virus genome quality result with each virus as a single input.

*06_dRep_outdir*: Result of dRep clustering. The directory contains the virus species clustering results for viruses that are assigned to the same genus.

*07_iPHoP_outdir*: Result of host prediction using iPHoP. The directory contains the iPHoP resulting folder(s) using the default iPHoP database, and custom MAGs from the same metagenome for virus identification if such custom MAGs are provided.

*08_ViWrap_summary_outdir*: Summarized results for viruses, including “Genus_cluster_info.txt” (virus genus clusters), “Species_cluster_info.txt” (virus species clusters), “Host_prediction_to_genome_m90.csv” (host prediction result at genome level; default confidence score cutoff as 90), “Host_prediction_to_genus_m90.csv” (host prediction result at genus level; default confidence score cutoff as 90), “Sample2read_info.txt” (reads counts and bases), “Tax_classification_result.txt” (virus taxonomy result), “Virus_annotation_results.txt” (virus annotation result), “Virus_genomes_files” (containing all “fasta,” “ffn,” and “faa” files for virus genomes), “AMG_results” (containing AMG statistics and protein sequences from all virus genomes), “Virus_raw_abundance.txt” (raw virus genome abundance), “Virus_normalized_abundance.txt” (normalized virus genome abundance; normalized by 100 M reads/sample), and “Virus_summary_info.txt” (summarized properties for all virus genomes, including genome size, scaffold number, protein count, AMG KOs, lytic/lysogenic state, CheckV quality, MIUViG quality, completeness, and completeness method).

*09_Virus_statistics_visualization*: Results of visualization of virus statistics. The directory contains two bar charts and two pie charts. The first bar chart represents the numbers of identified viral scaffolds, viruses, viral species, viral genera, viruses with taxonomy assigned, and viruses with hosts predicted. The second bar chart represents the relative abundance of AMG KOs. The first pie chart represents the relative abundance of virus families. The second pie chart represents the relative abundance of AMG KO metabolism. The raw inputs for visualization are also provided.

*ViWrap_run.log*: The log file. This file records the issued command and the time records of individual steps and the whole process.

By running the test data set representing the Guaymas Basin deep‐sea hydrothermal vent metagenome, we obtained 124 viral scaffolds that were binned into 91 viruses from the original 18,000 metagenomic scaffolds in the assembly. The total running time was ~14 h using 20 threads on a Ubuntu 18.04.6 LTS (×86_64) server. For the most time‐consuming parts, it took ~2 h to obtain viral scaffolds from metagenomic assemblies by both VIBRANT and VirSorter2, ~45 min to run vConTACT2 to cluster viral genomes, ~30 min to conduct host prediction by iPHoP using the default database, and ~10 h using custom MAGs as the database (making a new database takes longer as this process is limited by the phylogenetic tree building method implemented in iPHoP).

**Figure 3:**
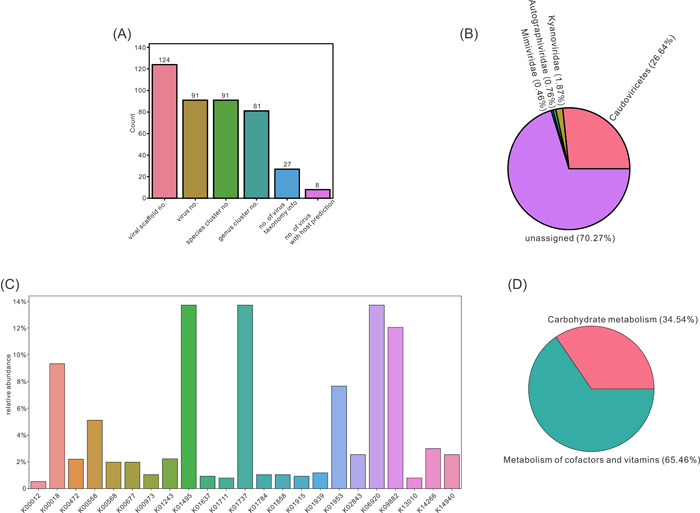
Visualizations of virus statistics. (A) Bar chart representing the numbers of identified viral scaffolds, viruses, viral species, viral genera, viruses with taxonomy assigned, and viruses with host predicted. (B) Pie chart representing the virus family relative abundance. (C) Bar chart representing the AMG KO relative abundance. (D) Pie chart representing the AMG KO metabolism relative abundance. AMG, auxiliary metabolic gene; KO, KEGG orthologs.

The visualized results based on virus statistics generally represent the findings of virus numbers, taxonomy, and AMG distribution (Figure 3). From 124 viral scaffolds, 91 viral genomes (including both binned and unbinned viruses) were reconstructed (Figure 3A). Each viral genome belonged to a distinct species and was further classified into 81 viral genera (Figure 3A). Within the 91 viral genomes, 27 had taxonomical classifications assigned, and 8 had hosts predicted (Figure 3A). With regard to the taxonomy, three families and one class were assigned with a summed virus relative abundance of around 29.7% (Figure 3B). There were 23 AMG KOs discovered in the viral community with their corresponding relative abundance fractions assigned (Figure 3C). When classifying KOs into KEGG metabolisms, two metabolisms, carbohydrate metabolism and cofactor and vitamin metabolism, were discovered to occupy the entire fraction (Figure 3D). The visualized results provided an intuitive and useful interpretation for general quantified features of the viral community, including virus numbers and statistics, virus family relative abundance, AMG KO relative abundance, and AMG KO metabolism relative abundance.

## DISCUSSION

ViWrap is a modular and comprehensive pipeline that integrates a full stream of virus analysis software/tools. ViWrap differs from previously developed software and tools, which only deal with a specific step of virus analysis, such as virus identification or virus binning. Compared to only conducting a specific “link” within the full “chain” of the analyses, an integrated tool is now critical for interpreting viral diversity and ecology. Significantly, ViWrap reduces the burden on users to benchmark and choose suitable software/tools for their analyses. As the study of uncultivated viral genomes from metagenomes becomes more important [2, 35], the standardized approach of ViWrap will enable the identification and analysis of viruses from metagenomes in a user‐friendly manner.

ViWrap integrates numerous state‐of‐the‐art mainstream and popular software/tools for virus analysis. It takes advantage of these component tools to achieve a comprehensive screening of viruses from metagenomes. ViWrap seamlessly connects the inputs and outputs of each step in the virus analysis pipeline with all the settings preconfigured. This feature saves time for users who may not have the necessary knowledge to choose suitable options and parameters for each tool used in the analysis. ViWrap also integrates the results from upstream and uses them for downstream analysis without having to conduct the same analysis again. For example, we use the vConTACT2 clustering analysis for both virus clustering and virus taxonomic classification steps. By putting the reference vOTU viruses (genus representative viruses) together with the query viruses and conducting the vConTACT2 clustering at the same time, users will save a considerable amount of time by integrating analyses for both steps.

The software offers users flexible options for custom usage, including the options to choose identification methods, utilize metagenomic reads (including both long reads and short reads), and incorporate custom MAGs from the same metagenome as an additional database for host prediction. Thus, ViWrap fits various application scenarios such as unraveling viral diversity and ecology in a microbiome or environment, identifying viruses and phage in metagenomes, identifying proviruses from publicly available genomes when genomic reads are inaccessible, discovering direct connections between viruses and MAGs reconstructed from the same metagenome, and so on. As viral sequences are generally shorter than microbial sequences, there is a growing need for long‐read sequencing technologies in viral sequencing and analysis. ViWrap's ability to generate coverage using long‐read sequencing data makes it well‐suited for these applications; therefore, facilitating viral sequencing and analysis in the future. ViWrap also provides comprehensive virus analysis results and visualized statistics that can be easily used for further downstream analysis and interpretation of results. The summary of statistics provided by ViWrap offers a comprehensive window into the viral community and viral ecological functions in a system.

Collectively, ViWrap is a one‐stop modular pipeline and wrapper that takes metagenome/virome and/or metagenomic reads as inputs and generates easy‐to‐read/parse virus analysis results in a user‐friendly, comprehensive, standardized (yet flexible for various application scenarios) manner. Although we demonstrate the application of ViWrap in a natural environment (hydrothermal vent environment in this study), the tools and databases implemented in ViWrap allow it to be widely used for various environments, such as man‐made environmental settings (i.e., industrial environment, wastewater treatment plants) and human‐microbiome‐related environmental settings (i.e., human body, human gastrointestinal tract, oral cavity). With the rapid growth of the field of viruses and phages in microbiomes, larger data sets and more advanced software/tools are being constantly developed and introduced. The modular nature of ViWrap will ensure easy integration of new tools and databases in the future. We propose that ViWrap has the potential to be widely adopted in the community and to standardize and advance the study of viruses in microbiomes.

## AUTHOR CONTRIBUTIONS

Zhichao Zhou and Karthik Anantharaman conceived the initial idea of ViWrap. Zhichao Zhou, Karthik Anantharaman, and Cody Martin contributed to the general function and framework of ViWrap. Zhichao Zhou and Cody Martin conducted the development and workflow of analyses. James C. Kosmopoulos contributed to the debugging process and the GitHub website. Karthik Anantharaman supervised this project. The manuscript was written by Zhichao Zhou and Karthik Anantharaman. All authors have read the final manuscript and approved it for publication.

## CONFLICT OF INTEREST STATEMENT

The authors declare no conflict of interest.

## Data Availability

Reconstructed genomes and metagenomic reads for the Guaymas Basin hydrothermal vent environment are available at NCBI BioProject PRJNA522654 and SRA SRR3577362. ViWrap is publicly accessible to all researchers on GitHub (https://github.com/AnantharamanLab/ViWrap) with detailed instructions. Supplementary Materials (figures, tables, scripts, graphical abstract, slides, videos, Chinese translated version, and updated materials) may be found in the online DOI or iMeta Science http://www.imeta.science/.
